# Differences in dietary patterns between native and immigrant populations in Spain

**DOI:** 10.1371/journal.pone.0328458

**Published:** 2025-07-21

**Authors:** Joaquín Moncho, Lauren Elena Ortega Sarabia, Eva María Trescastro-López, Alba Martínez-García

**Affiliations:** 1 Research Unit for the Analysis of Mortality and Health Statistics, Department of Community Nursing, Preventive Medicine, Public Health and History of Science, University of Alicante, Alicante, Spain; 2 Alicante Institute for Health and Biomedical Research (ISABIAL, Group 23), Alicante, Spain; 3 Alicante General University Hospital Dr. Balmis, Alicante, Spain; 4 Department of Nursing, University of Alicante, Alicante, Spain; 5 Balmis Research Group in History of Science, Health Care and Food, University of Alicante, Alicante, Spain; 6 Research Group on Applied Dietetics, Nutrition and Body Composition, University of Alicante, Alicante, Spain; 7 Department of Community Nursing, Preventive Medicine, Public Health and History of Science, University of Alicante, Alicante, Spain; Medical Research Council / Uganda Virus Research Institute & London School of Hygiene and Tropical Medicine Uganda Research Unit, UGANDA

## Abstract

**Background:**

Migration in Spain has increased in recent decades, leading to a multicultural society. These migrations have brought about a transcendental change in the lifestyles and health of migrants. The aim of these study was to describe the frequency of consumption of sugary drinks, fruit and vegetables in the native and immigrant population residing in the Valencian Community (Spain) and its possible relationship with certain socioeconomic characteristics and lifestyles.

**Methods:**

A representative sample of the population aged 15 years or older, non-institutionalised and resident in the Valencian Community. Descriptive cross-sectional observational study conducted on the basis of the Valencian Community Adult Health Survey, 2016. We analysed the consumption of fruit, vegetables and sugary soft drinks by country of birth and sex, and their possible association with socio-demographic characteristics and lifestyles.

**Results:**

5485 adults aged 15 years and older were analysed, 13.48% of whom were of immigrant origin. Overall, women had a significantly lower adjusted risk of inadequate vegetable consumption and lower than recommended fruit consumption. Men of immigrant origin showed a higher risk of lower than recommended vegetable consumption and a lower risk of inadequate consumption of sugar-sweetened soft drinks than natives. Women of immigrant origin showed a lower risk of lower-than-recommended vegetable consumption but a higher risk of inadequate consumption of sugar-sweetened soft drinks. Differences were also observed in the recommended consumption of these products according to educational level, social class, age, employment status, physical exercise and self-perceived weight.

**Conclusions:**

Public health policies and interventions should incorporate a population-based approach that takes into account the origin of the population and addresses social and economic inequalities, with an emphasis on the most at-risk groups.

## Introduction

In recent decades, there has been a notable increase in migration, which has contributed to the transformation of Spain into a multicultural country [1]. The National Statistics Institute (INE) reports that as of 1 January 2023, 8,204,206 individuals of immigrant origin (born abroad) reside in Spain, representing 17.06% of the total population. The main receiving regions are Catalonia (1,774,487), Madrid (1,535,784) and Valencia (1,091,230) [2].

These migrations entail a major change in the lifestyles of the people involved, who ultimately adopt the norms of the host country (acculturation). This process can have both positive and negative consequences for habits such as diet, physical activity or the consumption of toxic substances (drugs, alcohol, tobacco), among others [3–5].

The diets of migrants can be influenced by numerous socio-demographic factors such as gender, ethnicity, age, income, educational level, social status and geography that determine consumption patterns [4,6,7].

In this sense, consumption patterns refer to the amounts, proportions, combinations or varieties of foods and beverages consumed, as well as the frequency with which they are habitually consumed. Some consumption patterns have been identified as a significant risk factor for population health. According to the World Health Organization (WHO), the nutritional recommendations for a healthy population are 5 servings of fruits and vegetables per day and limiting their intake of foods and beverages with a high sugar content to the greatest extent possible [8]. The Mediterranean Diet is considered a healthy dietary pattern that meets these recommendations [9,10].In this regard, several studies have indicated a correlation between the consumption of sugar-sweetened beverages and an increased risk of developing type II diabetes mellitus, cardiovascular disease, dental caries and certain types of cancer [11–13]. In contrast, the consumption of fruits, vegetables, whole grains and other plant-based foods has been associated with a reduced risk of developing the aforementioned chronic diseases [8,14–17].

According to previous research in other countries [18–25], knowing the differences in the consumption patterns of sugary drinks, fruit and vegetables among migrant population compared to those born in the host country may facilitate the design of effective strategies to promote healthy eating habits in specific populations, taking cultural differences into account. This study is therefore necessary for several reasons. Spain is one of the countries with the largest immigrant population globally [26] comprising individuals from a multitude of nationalities and cultural backgrounds [1]. It is therefore evident that an understanding of the dietary patterns of the immigrant community is essential for the development of effective interventions and policies aimed at improving public health. Furthermore, there is a lack of research on this topic in Spain. For this reason, the present study is proposed.

The aim of the present study was to describe the frequency of consumption of sugar-sweetened beverages, fruit and vegetables among the native and immigrant population residing in the Valencian Community aged 15 years and older, and its possible relationship with certain socioeconomic characteristics and lifestyles.

## Methods

### Data sources

Descriptive cross-sectional observational study based on the Health Survey of the Valencian Community, corresponding to the year 2016, which included a representative sample of the population aged 15 years or older, non-institutionalised and resident in the said Community. The survey was carried out by the health authorities within the scope of the health policies of the Regional Ministry of Health of the Valencian Community [25]. The data collection was conducted by a company that had been awarded the contract between the months of May and December 2016.

Given the structure of the sample design used to collect the sample (complex sample design that assigned each subject a weighting factor according to its representativeness), all the analyses carried out took into account the corresponding weighting factors included in the databases provided by the Health Plans Service of the Regional Ministry of Health of the Generalitat Valenciana. Details on the survey methodology (sample design, sampling procedure, consent, ethics, etc.) have been published previously [27]. According to national regulations, the data from the National or Autonomous Health Surveys are public in Spain and the Valencian Health Authorities are responsible and guarantors of confidentiality and anonymity, so the approval of an ethical committee is not necessary [28]. The data provided to the study investigators are publicly available and anonymized, so this research does not raise ethical issues.

### Dependent variables

The dependent variables were consumption of fruit, vegetables and sugar-sweetened soft drinks. Initially, fruit and vegetable consumption were considered at three levels (less than one serving per day, one to two servings per day and three or more times per day). Consumption of sugary soft drinks was considered at five levels (one or more times a day, 4–6 times a week, 1–3 times a week, less than once a week and never). These variables were subsequently recoded twice in order to analyse, on the one hand, inadequate consumption of these products and, on the other hand, non-compliance with the recommendations on their consumption. The following were considered inadequate consumption of the different products analysed: not consuming fruit, not consuming vegetables or consuming them less than once a week, and consuming sugary drinks one or more times a day. Recommended consumption was considered to be consuming two or more fruits per day, three or more servings of vegetables per day and never consuming sugar-sweetened beverages, in line with the latest recommendations made for healthy populations [8,29,30].

### Explanatory variables

The main explanatory variables were: country of birth (Spain vs. others), sex (male, female) and age (age in years was considered as a continuous quantitative variable and categorized into 5 age groups: 15−24, 25−44, 45−64, 65−74 and 75 or more years), educational level (classified into four categories: University, Secondary, Primary and No studies), perception of weight (two classifications were considered: the first recoding included three categories: thin, normal and overweight/obesity; the second recoding included two categories: thin/normal and overweight/obesity), cohabitation in couples (Yes/No), employment status (was classified into three categories; working, unemployed, other), physical exercise (two classifications were considered: the first recoding included four categories: not at all, sometimes, regularly and several times a week; the second recoding included two categories: none vs. the rest) and social class based on the occupation. The social class categories considered were based on the classification proposed in 2012 by the Working Group on Determinants of the Spanish Society of Epidemiology (SEE), adapted for the ENSE [31]. These are obtained from the occupation, current or past, and coded to three digits according to the National Classification of Occupations that came into force in 2011 (CNO-11) [32]. The 6 categories proposed were: I. directors and managers of establishments with 10 or more employees and professionals traditionally associated with university degrees; II. directors and managers of establishments with less than 10 employees, professionals traditionally associated with university degrees, and other technical support professionals. Sportsmen and artists; III. intermediate occupations and self-employed workers; IV. supervisors and workers in skilled technical occupations; V. skilled primary sector workers and other semi-skilled workers; and VI. unskilled workers. For the analysis, these 6 categories were regrouped into 3 categories as follows: (1) Class I–II; (2) Class III–IV; and (3) Class V–VI.

### Data analysis

First, a descriptive analysis of the main study variables was carried out, disaggregating by country of birth (native/foreign) and sex (male/female). For categorical variables, frequency distribution tables were constructed. For the study of the associations between the variables related to the daily consumption of fruits and vegetables and sugar-sweetened beverages and the characteristics of physical condition, sociodemographic, socioeconomic, family and living habits, the Pearson chi-squared test, Fisher’s exact test or the Monte Carlo method were used, depending on the fulfilment of the conditions for the application of each of them for each of the population subgroups considered (natives or foreigners). Finally, multivariate logistic regression models were adjusted. The dependent variables in each case were: inadequate fruit consumption, inadequate vegetable consumption, inadequate consumption of sugar-sweetened beverages, and failure to comply with the recommendations for vegetables, fruits and sugar-sweetened beverages. The explanatory or independent variables included were, in addition to country of birth, sex, age, educational level, social class, cohabitation, self-perceived weight, employment status and leisure time physical exercise. Possible interaction and collinearity were taken into account in the adjustment of all models. The analysis was performed with the IBM SPSS Statistics v.25 statistical analysis program and a p value of less than 0.05 was considered significant.

## Results

The sample analysed included a total of 5485 individuals aged 15 years and over, resident in the Valencian Community, of whom 48.6% were men and 51.4% women, with percentages of people of immigrant origin of 14.2% and 12.8% respectively.

Significant differences (Table 1) were observed between the native population and those of immigrant origin by age (younger in the case of immigrants, p < 0.001), level of studies (higher level of studies in immigrants, p < 0.001), cohabitation as a couple (higher percentage of cohabitation in native and in immigrant women with p < 0.001 and p = 0.006, respectively), employment status (higher percentage of unemployed population in the case of immigrants, p < 0.001) and leisure time physical activity (higher physical activity in men of immigrant origin, p < 0.001 and lower in the case of women, p = 0.018) for both sexes. In addition, in men, significant differences were observed between natives and immigrants according to social class (higher in the case of the native population, p < 0.001) and weight perception (higher percentage of perceived weight higher than normal in natives, p < 0.001).

**Table 1:** Socio-demographic characteristics, physical activity and distribution of study subjects by sex, country of birth, socio-demographic characteristics and lifestyle.

	Male						Female					
	Country of birth	Spain		Other		Total		Spain		Other		Total	
		N	%	N	%	N	Sig	N	%	N	%	N	Sig
**Age**												
15-24 years	274	12.0	43	11.4	317	<0.001	267	10.9	38	10.5	305	<0.001
25-44 years	735	32.1	207	55.1	942		734	29.9	199	55.1	933	
45-64 years	766	33.5	110	29.3	876		808	32.9	92	25.5	900	
65-74 years	284	12.4	9	2.4	293		319	13.0	15	4.2	334	
≥75 years	229	10.0	7	1.8	236		329	13.4	17	4.7	346	
**Level of studies**												
No studies	230	10.1	27	7.2	257	<0.001	324	13.2	22	6.1	346	<0.001
Primary	667	29.2	57	15.2	724		671	27.4	60	16.7	731	
Secondary	943	41.2	200	53.3	1143		903	36.8	178	49.4	1081	
University	447	19.5	91	24.3	538		553	22.6	100	27.8	653	
**Cohabitation**												
Couples	1370	59.9	156	41.5	1526	<0.001	1218	49.6	207	57.3	1425	0.006
Others	918	40.1	220	58.5	1138		1236	50.4	154	42.7	1390	
**Social Class**												
Class I and II	514	23.3	36	9.7	550	<0.001	538	21.9	78	22.5	616	0.698
Class III and IV	779	35.4	125	33.8	904		811	33	130	37.6	941	
Class V and VI	909	41.3	209	56.5	1118		953	38.8	138	39.9	1091	
**Employment situation**												
Working	1027	45.0	195	51.9	1222	<0.001	838	34.5	178	49.3	1016	<0.001
Unemployed	325	14.2	97	25.8	422		408	16.8	93	25.8	501	
Other	933	40.8	84	22.3	1017		1185	48.7	90	24.9	1275	
**Leisure time physical activity**												
Nothing	598	26.5	60	16.5	658	<0.001	836	34.5	139	38.8	975	0.018
Sometimes	712	31.6	96	26.4	808		827	34.1	136	38.0	963	
Regularly	292	13.0	68	18.7	360		200	8.2	21	5.9	221	
Several a week	651	28.9	140	38.5	791		562	23.2	62	17.3	624	
**Weight perception**												
Thin	311	13.6	32	8.5	343	<0.001	356	14.5	48	13.3	404	0.598
Normal	1162	50.8	270	72.0	1432		1342	54.7	192	53.2	1534	
Overweight/Obesity	814	35.6	73	19.5	887		757	30.8	120	33.3	877	

Table 2 shows the frequencies of fruit and vegetable consumption. The most frequent consumption category for both fruit and vegetables were between 1–2 servings per day with total percentages of 44.9% and 60.6% respectively. This was also the case when disaggregated by country of birth. Significant differences in the frequency of daily fruit consumption were detected between natives and immigrants, both in men and women, especially in the categories of consumption of three or more servings and less than one serving per day. Native men had lower fruit consumption than immigrants (p < 0.001). In the case of women, it was the natives who had the highest fruit consumption (p < 0.001). In terms of vegetable consumption, native-born men had, overall, significantly lower vegetable consumption than those of immigrant origin with consumption percentages of less than one serving per day of 40% and 28.6% respectively (p < 0.001). In women, no significant differences in vegetable consumption were detected (p = 0.838).

**Table 2:** Frequency of fruit and vegetable consumption by sex and country of birth.

Daily servings	<1		1-2		≥3		Total	
	N	%	N	%	N	%	N	Sig
**Fruit servings**								
**Male**								
Spain	739	32.3	966	42.3	581	25.4	2286	<0.001
Others	101	26.9	137	36.4	138	36.7	376	
Total	840	31.6	1103	41.4	719	27.0	2662	
**Female**								
Spain	601	24.5	1146	46.8	703	28.7	2450	<0.001
Others	89	24.7	206	57.2	65	18.1	360	
Total	690	24.6	1352	48.1	768	27.3	2810	
**Total**								
Spain	1340	28.3	2112	44.6	1284	27.1	4736	0.363
Others	190	25.8	343	46.6	203	27.6	736	
Total	1530	27.9	2455	44.9	1487	27.2	5472	
**Vegetable servings**								
**Male**								
Spain	916	40.0	1202	52.6	169	7.4	2287	<0.001
Others	108	28.6	257	68.2	12	3.2	377	
Total	1024	38.4	1459	54.8	181	6.8	2664	
**Female**								
Spain	679	27.7	1616	65.9	156	6.4	2451	0.838
Others	100	27.8	240	66.7	20	5.5	360	
Total	779	27.7	1856	66.0	176	6.3	2811	
**Total**								
Spain	1595	33.7	2818	59.5	325	6.8	4738	<0.001
Others	208	28.2	497	67.4	32	4.4	737	
Total	1803	32.9	3315	60.6	357	6.5	5475	

Table 3 shows the frequency of consumption of sugary soft drinks. In general, natives showed a significantly lower consumption of these products (p < 0.001) than those of immigrant origin, both in men and in women. Forty per cent of natives never consumed this type of beverage compared to 34.1% of those of immigrant origin, while in women these percentages were 41.8% and 34.1% respectively. On the other hand, 23.7% of men and 24.1% of women of immigrant origin consumed sugary soft drinks 4 or more times a week, compared to 16.6% and 13.5% respectively in the case of natives. The daily consumption of these products was particularly high among women of immigrant origin compared to native women (21.6% vs. 8%).

**Table 3:** Frequency of consumption of sugar-sweetened soft drinks by sex and country of birth.

	Daily		4-6/week		1-3/week		<1/week		Never		Total	
	N	%	N	%	N	%	N	%	N	%	N	Sig
**Male**												
Spain	228	9.9	150	6.7	586	25.6	408	17.8	916	40.0	2288	<0.001
Others	28	7.5	61	16.2	84	22.3	75	19.9	128	34.1	376	
Total	256	9.6	211	7.9	670	25.2	483	18.1	1044	39.2	2664	
**Female**												
Spain	196	8.0	135	5.5	511	20.8	588	23.9	1026	41.8	2456	<0.001
Others	78	21.6	9	2.5	79	21.9	72	19.9	123	34.1	361	
Total	274	9.7	144	5.2	590	20.9	660	23.4	1149	40.8	2817	
**Total**												
Spain	424	8.9	285	6.1	1097	23.1	996	21.0	1942	40.9	4744	<0.001
Others	106	14.4	70	9.5	163	22.1	147	20.0	251	34.0	737	
Total	530	9.7	355	6.5	1260	23.0	1143	20.8	2193	40.0	5481	

Table 4 shows the frequency of fruit consumption according to socio-demographic characteristics and physical activity by sex and country of birth. The number of daily fruit servings was significantly associated with age (higher consumption in older adults), level of education (lower consumption the higher the level of education in natives and higher consumption the higher the level of education in immigrants), living with a partner (higher consumption if natives and women of immigrant origin live together and lower consumption if they live together in immigrant men), social class (higher consumption in social class I-II), employment status (higher consumption in social class I-II), employment status (higher consumption in immigrants) and the number of fruit servings per day (higher consumption in immigrants), social class (higher consumption in social class I-II), employment status (higher consumption in those who work or belong to the category of others, except in women of immigrant origin where there were no significant differences), physical activity (higher consumption if they exercise several times a week) and weight perception (higher consumption if they perceived themselves to be of normal weight. Lower consumption if they perceived themselves as overweight or obese in natives and men of immigrant origin or perceived themselves as thin in immigrant women), in both men and women.

**Table 4:** Frequency of fruit consumption according to socio-demographic characteristics and physical activity by sex and country of birth.

Sex	Male											Female										
Country of birth	Spain					Others						Spain					Others					
**Servings**		**<1**	**1-2**	**≥3**			**<1**	**1-2**	**≥3**				**<1**	**1-2**	**≥3**			**<1**	**1-2**	**≥3**		
	**N**	**%**	**%**	**%**	**sig**	**N**	**%**	**%**	**%**	**sig**	**sig**	**N**	**%**	**%**	**%**	**sig**	N	**%**	**%**	**%**	**sig**	**sig**
**Age**																						
15-24 years	274	42.7	51.1	6.2	<0.001	43	9.3	74.4	16.3	<0.001	<0.001	265	43.8	29.4	26.8	<0.001	38	52.6	44.7	2.6	<0.001	0.004
25-44 years	735	38.9	41	20.1		207	34.3	26.6	39.1		<0.001	734	32.7	51.6	15.7		199	22.6	57.8	19.6		0.02
45-64 years	767	35.6	35.9	28.6		110	17.3	40	42.7		<0.001	808	22.4	45.8	31.8		92	19.6	57.6	22.8		0.086
65-74 years	283	14.5	43.8	41.7		9	55.6	22.2	22.2		0.016	318	10.4	44.3	45.3		14	28.6	64.3	7.1		0.008
>75 years	234	10.3	55.6	34.2		7	28.6	57.1	14.3		0.163	326	9.8	54.6	35.6		17	11.8	70.6	17.6		0.198
**Level of study**																						
No studies	229	18.8	53.3	27.9	<0.001	28	35.7	42.9	21.4	<0.001	0.112	324	14.5	47.8	37.7	<0.001	22	36.4	45.5	18.2	0.008	0.015
Primary	666	33.6	37.8	28.5		56	16.1	37.5	46.4		0.005	667	25.2	47.5	27.3		60	18.3	51.7	30		0.498
Secondary	943	38.1	38.3	23.6		199	32.7	41.2	26.1		0.354	903	24.9	49.4	25.7		177	30.5	55.9	13.6		0.002
University	447	25.1	51.7	23.3		91	17.6	23.1	59.3		<0.001	552	28.3	41.3	30.4		100	15	65	20		<0.001
**Cohabitation in couples**																						
Yes	1368	30.5	39.5	30	<0.001	156	32.1	42.9	25	<0.001	0.429	1216	33.3	45.7	32.1	<0.001	207	22.7	53.1	24.2	<0.001	0.058
No	916	35	46.3	18.7		220	23.2	31.8	45		<0.001	1232	36.7	47.9	25.4		154	27.3	63	9.7		<0.001
**Social class**																						
I-II	514	26.5	41.6	31.9	<0.001	36	30.6	22.2	47.2	<0.001	0.056	538	18.8	46.1	35.1	<0.001	78	17.9	57.7	24.4	0.033	0.117
III-IV	778	34.6	46.4	19		125	20.8	56.8	22.4		0.009	809	17.2	51.9	30.9		131	19.8	64.9	15.3		0.001
V-VI	906	31.4	40.2	28.4		209	29.7	26.8	43.5		<0.001	951	34.8	42.8	22.4		138	31.9	50	18.1		0.254
**Employment situation**																						
Working	1026	39.1	35.9	25	<0.001	195	31.3	33.3	35.4	0.014	0.009	837	25	53.8	21.3	<0.001	177	23.2	56.5	20.3	0.797	0.797
Unemployed	325	39.7	38.8	21.5		96	31.3	35.4	33.3		0.053	408	32.1	38.5	29.4		92	25	59.8	15.2		<0.001
Others	930	22.2	50.6	27.2		84	11.9	44	44		0.002	1178	21.4	45.3	33.3		89	27	57.5	15.7		0.003
**Physical activity**																						
Nothing	595	35.5	35.6	28.9	<0.001	60	40	43.3	16.7	0.002	0.126	831	28.9	51.9	19.3	<0.001	139	28.8	59	12.2	0.005	0.113
Sometimes	711	33.5	46.4	20.1		95	29.5	36.8	33.7		0.01	827	24.3	49.1	26.6		136	17.6	61	21.3		0.034
Regularly	292	28.8	51	20.2		68	17.6	38.2	44.1		<0.001	199	14.6	46.7	38.7		20	15	80	5		0.007
Several a week	651	30	39.9	30.1		140	18.6	35.7	45.7		<0.001	559	19.7	37.2	43.1		62	30.6	41.9	27.4		0.032
**Weigh perception**																						
Thin	312	30.8	42.3	26.9	0.011	33	24.2	15.2	60.6	<0.001	<0.001	353	21.5	53	25.5	0.009	48	29.2	56.3	14.6	0.019	0.196
Normal	1160	29.7	44.9	25.4		270	28.1	34.4	37.4		<0.001	1340	23.5	45.6	30.9		191	24.1	51.8	24.1		0.135
Overweight/obesity	813	36.8	38.4	24.8		73	23.3	53.4	23.3		0.026	756	27.8	45.8	26.5		120	23.3	66.7	10		<0.001

In general, adults of immigrant origin consumed more servings of fruit than natives in age groups under 64 years, in those with at least primary education (in men), in those who do not live with a partner, in those belonging to social classes III-IV and V-VI, in those who work (in men), in those who do physical activity at least once a week and in those who perceive themselves to be slim or of normal weight in men and overweight or obese in women.

Table 5 shows the frequency of vegetable consumption according to socio-demographic characteristics and physical activity by sex and country of birth. The number of servings of vegetables per day was significantly associated with age (higher consumption in older adults), level of education (lower consumption in natives and women of immigrant origin with secondary education and with primary education in immigrant men), living with a partner (higher consumption if they live together in immigrant men), social class (higher consumption in social class I-II), employment status (lower consumption in immigrant men), social class (higher consumption in social class I-II), employment status (lower consumption in unemployed natives and in those who work in immigrants), physical activity (lower consumption the less physical activity), and weight perception (higher consumption if they perceived themselves to be of normal weight, except in men of immigrant origin where they had lower consumption).

**Table 5:** Frequency of vegetable consumption by socio-demographic characteristics and physical activity by sex and country of birth.

Sex	Male											Female										
Country of birth	Spain					Others						Spain					Others					
**Servings**		**<1**	**1-2**	**≥3**			**<1**	**1-2**	**≥3**				**<1**	**1-2**	**≥3**			**<1**	**1-2**	**≥3**		
	**N**	**%**	**%**	**%**	**sig**	**N**	**%**	**%**	**%**	**sig**	**sig**	**N**	**%**	**%**	**%**	**sig**	**N**	**%**	**%**	**%**	**sig**	**sig**
**Age**																						
15-24 years	274	39.8	55,1	5.1	<0.001	43	9.3	88.4	2.3	<0.001	<0.001	266	46.6	51.1	2.3	<0.001	38	42.1	57.9	0	<0,001	0.523
25-44 years	735	48.2	42	9.8		207	42.5	56	1.4		<0.001	734	34.9	58.4	6.7		200	29	66	5		0.149
45-64 years	766	40.2	51,8	8		110	11.8	81.8	6.4		<0.001	808	19.9	72.6	7.4		93	21.5	74.2	4.3		0.528
65-74 years	284	25.7	70,4	3.9		9	11.1	88.9	0		0.615	317	17	75.7	7.3		15	26.7	33.3	40		<0.001
>75 years	226	31.4	63,7	4.9		7	14.3	71.4	14.3		0.291	327	26	68.5	5.5		16	12.5	82.3	6.3		0.462
**Level of study**																						
No studies	229	34.1	62,4	3.5	<0.001	27	33.3	59.3	7.4	<0.001	0.609	322	24.5	68.9	6.6	<0.001	22	22.7	77.3	0	<0,001	0.434
Primary	666	37.4	58	4.6		57	49.1	47.4	3.5		0.216	668	26	67.4	6.6		61	18	70.5	11.5		0.182
Secondary	944	44.5	45,1	10.4		200	31.5	65.5	3		<0.001	903	32.8	61	6.2		178	36.5	55.6	7,9		0.372
University	448	37.7	55,1	7.2		92	7.6	90.2	2.2		<0.001	553	22.4	71.3	6.3		90	19.2	80.8	0		0.021
**Cohabitation in couples**																						
Yes	1369	38.4	54,4	7.2	0.073	156	25	66.6	6.4	0.008	0.003	1218	24.8	67.7	7.5	0.328	207	23.7	71.5	4.8	0,521	<0.001
No	918	42.7	49,7	7.6		220	30.9	68.2	0.9		<0.001	1332	30.4	64.3	5.3		152	33.6	59.8	6.6		0.024
**Social class**																						
I-II	514	29.2	59,9	10.9	<0.001	5	11.1	86.1	2.8		0.007	539	21.5	72.2	6.3	<0.001	27	21.8	65.4	12.8		0.106
III-IV	779	43.1	51,6	5.3		39	30.4	68.8	0.8		<0.001	810	22.7	71.4	5.9		45	30.8	65.4	3.8		0.107
V-VI	907	41	51,4	7.6		75	31.1	64.1	4.8		0,004	951	34.2	59	6.8		46	29	66.7	4.3		0.192
**Employment situation**																						
Working	1027	41.1	48	10.9	<0.001	75	35.2	61.7	3.1		<0.001	837	25.6	66.9	7.5	<0.001	68	33.1	61.8	5.1		0.083
Unemployed	325	56.3	37,8	5.8		19	16.7	80.2	3.1		<0.001	407	37.6	52.8	9.6		22	21.5	76.3	2.2		<0.001
Others	932	33	62,8	4.2		25	27.7	69.9	2.4		0.393	1180	24.9	70.6	4.5		32	24.4	64.4	11.1		0.02
**Physical activity**																						
Nothing	598	47.8	48,5	3.7	<0.001	28	38.3	53.3	8.3		0.123	832	32,3	63,3	4,3	<0,001	39	26,6	71,9	1,4		0,077
Sometimes	712	37.5	55,6	6.9		32	28.1	66.7	5.2		0.122	827	29,7	64	6,3		55	30,1	59,6	10,3		0,212
Regularly	293	45.4	47,4	7.2		13	19.1	80.9	0		<0.001	199	14,6	81,9	3,5		7	25	65	10		0,106
Several a week	651	33.6	55	11.4		36	24.8	74.5	0.7		<0.001	461	20,7	69	10,3		16	22,6	74,2	3,2		0,197
**Weigh perception**																						
Thin	311	41.8	50,5	7.7	<0.001	2	6.3	93.8	0		<0.001	355	33,8	60,8	5,4	<0,001	15	31,3	68,8	0		0,214
Normal	1160	36.3	57	6.7		98	33.8	63.6	2.6		0.016	1339	25	69,2	5,8		55	21,5	71,2	7,3		0,438
Overweight/obesity	813	44.8	47	8.2		18	18.9	75.7	5.4		<0.001	757	29,6	62,5	7,9		50	36,4	58,7	5		0,219

In general, adults of immigrant origin consumed more vegetable servings than natives in age groups below 64 years, in those with at least secondary education, in those not living with a partner (in men), in all social classes, in those who were working (in men) or unemployed, in those who were physically active at least regularly (in men) and in all categories of weight perception in men.

Table 6 shows the frequency of consumption of sugar-sweetened soft drinks according to socio-demographic characteristics and physical activity by sex and country of birth. In general, higher consumption of this product was significantly associated with younger age, higher level of education, not living with a partner, lower social class, being unemployed, lower frequency of physical exercise (except for women of immigrant origin with higher consumption among those who exercised regularly), and an overweight/obese weight perception, both in men and women. Significant differences in the frequencies of consumption of sugary soft drinks between natives and immigrants were detected in almost all categories of each of the characteristics analysed, in both men and women. In general, adults of immigrant origin consumed more sugary soft drinks than natives in those under 24 years of age, in those with secondary or lower education in men and secondary or university education in women, in all categories of cohabitation, in all social classes except I-II, in all categories of employment status except those who were unemployed in men, in all levels of physical activity in women and in all categories of weight perception in women.

**Table 6:** Frequency of consumption of sugar-sweetened soft drinks according to socio-demographic characteristics and physical activity by sex and country of birth.

Sex	Male															Female														
Country of birth	Spain							Others								Spain							Others							
**Weekly frequency of soft drink consumption**	**N**	**≥7**	**4-6**	**1-3**	**<1**	**No**	**sig**	**N**	**≥7**	**4-6**	**1-3**	**<1**	**No**	**sig**	**sig**	**N**	**≥7**	**4-6**	**1-3**	**<1**	**No**	**sig**	**N**	**≥7**	**4-6**	**1-3**	**<1**	**No**	**sig**	**sig**
**Age**																														
15-24 years	275	8.7	19.6	42.2	13.1	16.4	<0.001	42	16.7	61.9	9.5	2.4	9.5	<0.001	<0.001	266	12.8	15	32.7	22.2	17.3	<0.001	38	28.9	7.9	13.2	36.8	13.2	<0.001	0.006
25-44 years	735	16.1	9.1	39.6	17.8	17.4		208	6.7	12.5	21.6	17.3	41.8		<0.001	734	11.4	6.7	29.7	25.2	27		200	25	2.5	25.5	16	31		<0.001
45-64 years	766	8.9	2.5	17.5	20.1	51		110	6.4	8.2	30.9	30.9	23.6		<0.001	807	6.9	4.7	17.5	27.1	43.7		91	16.5	0	20.9	26.4	36.3		0.004
65-74 years	284	3.9	1.8	10.9	20.1	63.4		9	0	0	0	33.3	66.7		0702	320	1.9	0.3	13.1	21.6	63.1		14	0	7.1	21.4	0	71.4		0.029
>75 years	228	3.1	1.8	6.6	13.2	75.4		90	0	0	0	14.3	85.7		1	328	4.6	2.1	7	17.1	69.2		17	5.9	0	5.9	11.8	76.5		0.92
**Level of study**																														
No studies	229	5.7	3.1	10.5	13.1	67.7	<0.001	27	7.4	0	33.3	7.4	51.9	<0.001	0.031	324	5.9	2.8	9.6	16	65.7	<0.001	22	22.7	0	22.7	4.5	50	<0.001	0.01
Primary	667	11.5	3.9	21.4	19.8	43.3		56	3.6	5.4	30.4	46.4	14.3		<0.001	669	8.5	3.1	15.4	24.8	48.1		60	6.7	1.7	18.3	13.3	60		0.236
Secondary	943	12.9	6.5	27.3	17.4	35.9		198	9.6	28.8	14.6	17.2	29.8		<0.001	903	9.5	8.9	23.4	24.5	33.8		177	23.7	4.5	18.6	26	27.1		<0.001
University	447	3.6	12.3	36.5	18.1	29.5		90	4,.4	0	31.1	13.3	51.1		<0.001	553	6	4.7	30	26.4	32.9		100	27	0	30	16	27		<0.001
**Cohabitation in co** **uples**																														
Yes	1370	7.9	2.9	18.8	19.1	51.2	<0.001	155	11	10.3	27.7	20	31	0.006	<0.001	1218	5.7	3.8	17.8	27.5	45.2	<0.001	207	16.4	1.9	24.6	16.9	40.1	0.002	<0.001
No	919	13.1	12	35.8	15.9	23.3		220	5	20.5	18.2	20	36.4		<0.001	1235	10.3	7.2	23.8	20.2	38.5		154	28.6	3.9	17.5	24	26		<0.001
**Social class**																														
I-II	515	4.5	3.9	34.6	16.9	40.2	<0.001	36	5.6	0	44.4	30.6	19.4	<0.001	0.037	538	6.5	3.5	25.7	24.5	39.8	<0.001	78	5.1	1.3	30.8	20.5	42.3	<0.001	0.642
III-IV	778	9	9.8	22.6	16.5	42.2		125	1.6	27.2	27.2	25.6	18.4		<0.001	811	4.8	8.6	21	26.6	39		129	30.2	2.3	12.4	14.7	40.3		<0.001
V-VI	909	12	4.3	23.3	20.9	38.7		209	11	12.4	16.3	14.8	45.5		<0.001	953	12.2	3.8	19.5	21.7	42.8		138	24.6	3.6	23.9	25.4	22.5		<0.001
**Employment situation**																														
Working	1026	9.6	4.6	28.4	23.6	33.9	<0.001	195	7.2	14.4	15.9	13.8	48.7	<0.001	<0.001	838	5.3	7	26	27.6	34.1	<0.001	177	22	1.7	24.9	15.3	36.2	0.004	<0.001
Unemployed	325	20.6	9.2	35.7	9.5	24.9		96	7.3	6.3	43.8	25	17.7		<0.001	408	17.6	6.4	17.6	24	34.3		93	22.6	0	23.7	29	24.7		0.024
Others	932	6.8	7.6	19.1	14.4	52.1		84	8.3	32.1	13.1	28.6	17.9		<0.001	1183	6.7	4.1	18.3	21.3	49.6		90	18.9	7.8	14.4	18.9	40		<0.001
**Physical activity**																														
Nothing	598	19.9	5	18.2	16.2	40.6	<0.001	60	10	23.3	30	6.7	30	<0.001	<0.001	834	7.8	6.4	17.3	23.7	44.8	<0.001	140	19.3	5	25	19.3	31.4	<0.001	<0.001
Sometimes	712	5.8	6.7	22.5	20.8	44.2		96	6.3	7.3	19.8	28.1	38.5		0.544	826	9.7	5.3	20.1	25.7	39.2		137	24.8	1.5	25.5	16.8	31.4		<0.001
Regularly	292	11	12.3	26	21.6	29.1		69	5.8	5.8	26.1	29	33.3		0.257	200	11	8.5	35	75	38		21	47.6	4.8	0	28.6	19		<0.001
Several a week	651	5.4	5.7	34.4	15.2	39.3		141	9.2	24.8	14.2	17	34.8		<0.001	562	4.3	3.9	22.4	27.4	42		62	11.3	0	11.3	25.8	51.6		0.016
**Weigh perception**																														
Thin	311	11.3	3.2	26.7	14.5	44.4	<0.001	33	6.1	12.1	3	54.5	24.2	<0.001	<0.001	355	7.6	11.3	15.8	24.2	41.1	<0.001	48	27.1	0	0	33.3	39.6	<0.001	<0.001
Normal	1161	6.8	10.3	28.3	15.2	39.4		270	7.8	19.6	21.9	18.9	31.9		<0.001	1341	6.2	5.1	24.5	13.3	40.9		191	16.2	3.7	24.1	18.3	37.7		<0.001
Overweight/obesity	814	13.8	2.5	21.4	23	39.4		73	6.8	6.8	31.5	8.2	46.6		<0.001	757	11.2	3.6	16.6	25	43.6		120	27.5	1.7	27.5	17.5	25.8		<0.001

### Multivariate analysis

Table 7 presents the results of the multivariate analysis. Higher educational attainment was significantly associated with a lower risk of inadequate vegetable consumption (OR=0.20 in university students, p < 0.001; OR=0.32, p < 0.001 in high school vs. no education) and sugary soft drinks (OR=0.55, p = 0.017 in university students), a lower risk of lower than recommended fruit consumption (OR=0.69 in university students, p = 0.007; OR=0.68, p = 0.002 in high school students) and a higher risk of higher than recommended consumption of sugary drinks (OR=1.44, p = 0.009 in university students). A lower level of social class was significantly associated with a lower risk of inadequate vegetable consumption (OR=0.43, p < 0.001 social class III-IV vs I-II) and a higher risk of inadequate consumption of sugar-sweetened soft drinks (OR=1.86, p < 0.001 social class V-IV vs I-II), lower than recommended fruit (OR=1.34, p < 0.001 social class III-IV vs I-II) and vegetable (OR=1.91, p < 0.001) consumption. Being unemployed was significantly associated with a higher risk of inadequate (OR=2.18, p < 0.001) and higher than recommended (OR=1.34, p = 0.002) consumption of soft drinks with sugar. Similarly, not living with a partner was significantly associated with an increased risk of inadequate (OR=1.29, p = 0.027) and higher than recommended (OR=1.38, p < 0.001) consumption of soft drinks with sugar (OR=1.38, p < 0.001).

**Table 7:** Adjusted odds ratios for inadequate and recommended intakes of fruit, vegetables and soft drinks containing sugar.

Variable	Inadequate fruit consumption		Inadequate vegetable consumption		Inadequate (excessive) consumption of sugar-sweetened soft drinks		Lower than recommended fruit consumption		Lower than recommended vegetable consumption		Higher than recommended consumption of sugar-sweetened soft drinks	
	OR (IC 95%)	Sig	OR (IC 95%)	Sig	OR (IC 95%)	Sig	OR (IC 95%)	Sig	OR (IC 95%)	Sig	OR (IC 95%)	Sig
**Sex**												
Male	1.15 (0.63;2.10)	0.647	0.53 (0.39;0.74)	<0,001	0.80 (0.64;0.99)	0.047	0.85 (0.76;0.96)	0.007	1.04 (0.82;1.32)	0.73	0.99 (0.88;1.13)	0.974
Female	1		1		1		1		1		1	
**Education**		0.254		<0,001		0.021		0.012		<0.001		0.026
University	0.47 (0.12;1.77)	0.263	0.20 (0.101;0.410)	<0,001	0.55 (0.34;0.90)	0.017	0.69 (0.52;0.90)	0.007	1.17 (0.66;2.07)	0.588	1.44 (1.106;1.90)	0.009
Secondary	0.65 (0.21;1.99)	0.448	0.32 (0.18;0.57)	<0,001	0.73 (0.47;1.13)	0.160	0.68 (0.53;0.87)	0.002	0.66 (0.39;1.09)	0.106	1.20 (0.94;1.53)	0.148
Primary	0.35 (0.11;1.11)	0.075	0.90 (0.54;1.50)	0,695	0.90 (0.59;1.38)	0.639	0.80 (0.63;1.01)	0.066	0.93 (0.57;1.54)	0.794	1.31 (1.04;1.66)	0.023
No studies	1		1		1		1		1		1	
**Social class**		0.054		<0,001		<0.001		<0.001		<0.001		0.391
V-VI	2.99 (1.06;8.43)	0.038	0.91 (0.60;1.38)	0,658	1.86 (1.37;2.55)	<0.001	1.10 (0.92;1.30)	0.291	1.22 (0.907;1.65)	0.208	1.01 (0.84;1.21)	0.904
III-IV	1.65 (0.55;4.91)	0.368	0.41 (0.25;0.67)	<0,001	1.22 (0.88;1.68)	0.231	1.34 (1.13;1.58)	<0.001	1.91 (1.40;2.62)	<0.001	1.10 (0.92;1.31)	0.279
I-II	1		1		1		1		1		1	
**Employment situation**		0.052		0,078		<0.001		0.002		<0.001		0.007
Others	1.07 (0.55;2.11)	0.834	0.77 (0.53;1.14)	0,191	1.02 (0.78;1.32)	0.847	0.80 (0.69;0.92)	0.002	2.46 (1.81;3.34)	<0.001	1.11 (0.95;1.30)	0.186
Unemployed	0.11 (0.02;0.69)	0.019	1.27 (0.84;1.93)	0,255	2.18 (1.71;2.79)	<0.001	1.03 (0.88;1.22)	0.690	1.32 (0.97;1.81)	0.079	1.34 (1.12;1.61)	0.002
Working	1		1		1		1		1		1	
**Country of birth**												
Others	0.40 (0.13;1.23)	0.111	0.61 (0.36;1.03)	0,066	0.642 (0.42;0.99)	0.045	0.90 (0.76;1.07)	0.225	2.75 (1.46;5.15)	0.002	0.88 (0.74;1.06)	0.178
Spain	1		1		1		1		1		1	
**Cohabitation in co** **uples**												
No	0.80 (0.40;1.61)	0.530	1.04 (0.74;1.47)	0,815	1.29 (1.03;1.61)	0.027	0.95 (0.83;1.08)	0.401	0.92 (0.72;1.18)	0.516	1.38 (1.20;1.57)	<0.001
Yes	1		1		1		1		1		1	
**Age**	0.97 (0.95-0.99)	0.002	0.98 (0.97;0.99)	<0,001	0.96 (0.96;0.97)	<0.001	0.97 (0.96;0.97)	<0.001	0.99 (0.98;0.99)	0.003	0.95 (0.95;0.96)	<0.001
**Weight perception**												
Overweight/obesity	1.21 (0.63;2.32)	0.561	0.98 (0.70;1.36)	0,898	1.89 (1.53;2.33)	<0.001	1.40 (1.23;1.59)	<0.001	0.78 (0.62;0.98)	0.041	1.30 (1.13;1.48)	<0.001
Thin/Normal	1		1		1		1		1		1	
**Physical exercise**												
No	1.99 (1.07;3.70)	0.030	1.57 (1.14;2.16)	0,006	1.80 (1.46;2.27)	<0.001	1.85 (1.62;2.12)	<0.001	1.98 (1.48;2.65)	<0.001	1.28 (1.11;1.48)	0.001
Yes	1		1		1		1		1		1	
Sex * Country of birth (Inmigrant woman)					4.58 (2.68;7.81)	<0.001			0.44 (0.20;0.98)	0.045		

Age was significantly associated with the consumption of all products, so that the older the age, the lower the risk of inadequate consumption or of not reaching the consumption recommendations for the different products analysed. Being overweight or obese was significantly associated with a higher risk of inadequate consumption of soft drinks with sugar (OR=1.89, p < 0.001), lower than recommended consumption of fruit (OR=1.40, p < 0.001) and higher than recommended consumption of soft drinks with sugar (OR=1.30, p < 0.001). Vegetable consumption was associated with a lower risk of lower than recommended consumption (OR=0.78, p = 0.041). Not exercising at all was significantly associated with a higher risk of inadequate intake or not reaching the consumption recommendations for all the products analysed.

Being female was significantly associated with a lower risk of inadequate vegetable consumption (OR=0.53, p < 0.001) and lower than recommended fruit consumption (OR=0.85, p = 0.007). A significant interaction was detected between sex and country of birth for inadequate consumption of soft drinks with sugar (p < 0.001) and recommended vegetable consumption (p = 0.045). Thus, while men of immigrant origin had a higher risk of lower than recommended vegetable consumption (OR=2.75) and a lower risk of inadequate consumption of sugar-sweetened soft drinks (OR=0.64) than natives, women of immigrant origin showed a lower risk of lower than recommended vegetable consumption (OR=1.04 x 0.44 = 0.464) but a higher risk of inadequate consumption of sugar-sweetened soft drinks (OR=0.80 x 4.58 = 3.66).

## Discussion

The results obtained show differences in the consumption patterns of fruit, vegetables and soft drinks with sugar between the native and immigrant populations. A higher consumption of fruit was observed in adults of immigrant origin than in natives for both sexes, as well as a higher risk of not reaching the recommended daily intake of vegetables. These results partially coincide with those obtained in a similar study in Portugal [18], where immigrants consumed a higher proportion of fruit and vegetables than natives. Other studies have found a higher risk of inadequate fruit and vegetable consumption in immigrants of different nationalities compared with the native population [5,25,33]. The scarcity or unavailability of certain types of vegetables that are part of the immigrants’ traditional diet, as well as lack of knowledge about the new foods of the host country and how to prepare them, could be some of the reasons for this trend [21]. Additionally, men had lower fruit and vegetable consumption than women, regardless of country of birth, as shown in other studies [5,18,34,35]. This finding may reflect gender differences in health consciousness and awareness of the need for a healthy diet. In previous research, masculine roles were associated with men’s preferences for meat, energy-dense foods and alcohol consumption (such as beef burgers, fries, pizza and beer) [36]. This finding suggests that gender can be used as a strategic factor in FV promotion for both sexes, promoting vegetables and plant proteins that appeal to men by linking the benefits of these foods to masculinity goals [35].

Likewise, consumption of sugar-sweetened soft drinks was higher in immigrants compared to natives, both men and women, a finding shared by several studies where migrant were more likely to consume sugary drinks [21,37]. In this sense, the inappropriate consumption of sugar-sweetened soft drinks was more frequent in younger age groups, independent of the other study variables. Considering that adults of immigrant origin were predominantly young in this research, it is possible that this tendency is one of the explanations for the higher consumption of sugar-sweetened soft drinks by this population. Several studies show similar results, with younger adults being more likely to consume these beverages, and young adults being four times more likely to consume them regularly [12,38].^,^ One explanation for this could be that adolescents and young adults may be more susceptible to media advertising of sugar-sweetened soft drinks, as well as their low market prices, making them a more attractive and affordable option for them. On the other hand, it is important to note that women of immigrant origin were found to be the highest consumers of sugar-sweetened beverages and to be at the highest risk of inappropriate consumption of sugar-sweetened beverages, which is interesting because in other studies it was immigrant men who were the highest consumers [22,37]. Therefore, it would be interesting to study this fact in this context if there is a gender issue involved, such as aspects of differentiated social integration.

In addition, it was also found that the higher the age (regardless of country of birth), the lower the risk of under-consuming or not meeting the recommended intake of fruit, vegetables and sugar-sweetened soft drinks. These results are in line with previous studies [18,38]. This may be because older people tend to have healthier eating habits, better cooking skills and more time to cook.

Higher levels of education were associated with a lower adjusted risk of inadequate vegetable consumption and lower than recommended fruit consumption, regardless of country of birth. These results are consistent with those obtained in other studies in Europe [5,18,34] and Thailand [35], where people with secondary and university education had better fruit and vegetable intake [39]. This could be explained by the fact that educational level may be associated with a higher level of nutritional knowledge [35], which in turn tends to be associated with favourable attitudes, greater interest and concern towards healthy eating habits. Furthermore, higher levels of education are often associated with higher levels of income, which may make it easier for this group to access healthy food.

Besides, higher risks of underconsumption of vegetables and inadequate consumption of sugar-sweetened soft drinks were found in adults from lower social classes. The association between social class and diet is similar to that observed in other studies [40,41], as lower economic status makes it more difficult to maintain access to healthy foods and may increase consumption of more affordable food options, such as fast food and sugary soft drinks, which are often low in essential nutrients and high in calories [37]. Furthermore, not living with a partner was associated with a higher adjusted risk of inadequate consumption of sugar-sweetened soft drinks [42], which may increase the risk of several diseases, such as type 2 diabetes or various types of cancer [43,44]. In this case, native-born men and foreign-born women living alone reported inadequate diet more frequently than their counterparts from different countries of birth. According to previous research [40], men tend to adopt the style of eating that is considered best for the family and the fact of living with a woman, who is traditionally associated with preparing meals at home, would be associated with better eating habits. Foreign women living alone may be more likely to neglect healthy food choices on a daily basis due to a lack of motivation to take care of the family’s diet, as well as factors related to adapting to a new country.

In terms of employment status, being unemployed was associated with twice the adjusted risk of inadequate consumption of sugar-sweetened soft drinks and a higher risk of not meeting vegetable consumption recommendations compared with those who were employed; also being retired, studying or doing housework was also associated with twice the risk of eating fewer vegetables than recommended. These results are in line with other studies [18], which suggest that those who work have a higher consumption of vegetables and fruit. This could be due to the fact that not working is associated with limited purchasing power, which may lead individuals to opt for cheap and unhealthy diets [40,45]. This finding is important because policies and/or strategies aimed at improving the nutritional status of specific groups should take these issues into account and not just focus on the food issue itself.

Interestingly, self-perceived weight also plays an important role in dietary food choices. In this case, it was observed that those who perceived themselves as overweight or obese had a lower risk of under-consumption of vegetables. This may be due to the fact that as the perception of being overweight influences the dietary pattern by increasing the intake of vegetables. This observation of the influence of self-perceived weight on dietary patterns has been described in other studies [46].

Regarding exercise, lack of physical activity was associated with a lower quality diet, with a higher risk of inadequate consumption of fruit, vegetables and sugary soft drinks and of not meeting the respective consumption recommendations, as seen in other studies [18,25,47]. This could be explained by the fact that, in general, people who are physically active are more likely to be interested in a healthy diet as part of a healthy lifestyle.

### Limitations

The present study has some limitations that should be considered when interpreting its results. Firstly, it should be noted that, as is well known, the observational and cross-sectional design of the study does not allow us to establish causal relationships, and is limited to identifying significant associations between the variables of interest.

On the other hand, given that some years have passed since the data used in the study were collected, the results obtained may not reflect the reality of current dietary patterns in the population. In addition, although the data collection was carried out by trained interviewers, the difficulty of respondents to accurately recall the amounts and types of food, as well as the influence of emotional and social factors, could affect the accuracy of the information collected.

Finally, the variables fruit portions consumed per day and vegetable portions consumed per day have been treated separately in this study, which has made it difficult to compare with the majority of studies, in which fruit and vegetables are grouped together to study their consumption. However, we believe that this distinction provided a more detailed study of the consumption of each food group, setting a recommended threshold for each and facilitating future recommendations.

## Conclusions

There are differences in the consumption patterns of fruit, vegetables and sugary drinks between the native and immigrant population of the Valencian Community. Knowing the socio-demographic variables that can lead to unhealthy eating patterns, such as being unemployed, not living with a partner, having a low level of education, belonging to a lower level of social class or not taking physical exercise, allows us to implement appropriate actions to intervene and improve the diet of the immigrant population by adapting strategies and policies to each population group and their needs. Therefore, public health policies and interventions aimed at the general population should incorporate an approach that takes into account the origin of the population and addresses social and economic inequalities, with an emphasis on these higher-risk groups.
